# How measurements affected by medication use are reported and handled in observational research: A literature review

**DOI:** 10.1002/pds.5437

**Published:** 2022-05-04

**Authors:** Jungyeon Choi, Olaf M. Dekkers, Saskia le Cessie

**Affiliations:** ^1^ Department of Clinical Epidemiology Leiden University Medical Center Leiden The Netherlands; ^2^ Department of Clinical Epidemiology & Department of Endocrinology and Metabolism Leiden University Medical Center Leiden The Netherlands; ^3^ Department of Clinical Epidemiology & Department of Biomedical Data Sciences Leiden University Medical Center Leiden The Netherlands

**Keywords:** intercurrent events, measurement error, medication use, research question, selection bias

## Abstract

**Purpose:**

In epidemiological research, measurements affected by medication, for example, blood pressure lowered by antihypertensives, are common. Different ways of handling medication are required depending on the research questions and whether the affected measurement is the exposure, the outcome, or a confounder. This study aimed to review handling of medication use in observational research.

**Methods:**

PubMed was searched for etiological studies published between 2015 and 2019 in 15 high‐ranked journals from cardiology, diabetes, and epidemiology. We selected studies that analyzed blood pressure, glucose, or lipid measurements (whether exposure, outcome or confounder) by linear or logistic regression. Two reviewers independently recorded how medication use was handled and assessed whether the methods used were in accordance with the research aim. We reported the methods used per variable category (exposure, outcome, confounder).

**Results:**

A total of 127 articles were included. Most studies did not perform any method to account for medication use (exposure 58%, outcome 53%, and confounder 45%). Restriction (exposure 22%, outcome 23%, and confounders 10%), or adjusting for medication use using a binary indicator were also used frequently (exposure: 18%, outcome: 19%, confounder: 45%). No advanced methods were applied. In 60% of studies, the methods' validity could not be judged due to ambiguous reporting of the research aim. Invalid approaches were used in 28% of the studies, mostly when the affected variable was the outcome (36%).

**Conclusion:**

Many studies ambiguously stated the research aim and used invalid methods to handle medication use. Researchers should consider a valid methodological approach based on their research question.

**Key Points**

- Methodological studies stressed the importance of adequately handling variables affected by medication use and showed that using invalid methods may lead to substantial bias. However, we found that many clinical studies did not consider this issue.
- A large proportion of the studies did not provide information on whether their interest was in the observed or the untreated underlying values. Without clear reporting on research aims, interpretation of the results will be ambiguous.
- Methods which have been shown invalid, such as restricting a study population to non‐medication users when the outcome variable was affected by medication use, are still often used.
- Justification on methods used for handling medication use was seldom given.

**Plain Language Summary**

In epidemiological research, measurements affected by medication, for example, blood pressure lowered by antihypertensives, are common. Different ways of handling medication are required depending on the research questions and the function of the measurement in the analysis (e.g., whether the blood pressure is the exposure or the outcome). Incorrect handling of medication use in the analysis may introduce bias or lead to a wrong interpretation of the result. This study aimed to review how medication use are being handled in observational research. We review a total of 127 articles. Most studies did not perform any method to account for medication use and no advanced methods were applied. In 60% of studies, the validity of the method used could not be judged due to ambiguous reporting of the research aim. Invalid approaches were used in 28% of the studies. Many studies ambiguously stated the research aim and used invalid methods to handle medication use. We urge researchers to consider a valid methodological approach based on their research question.

## 1 INTRODUCTION

Measurements affected by medication use are a commonly encountered feature in epidemiological research. For example, blood pressure is lowered by antihypertensive drugs or glucose levels by glucose‐lowering drugs. Several methods for handling medication use have been proposed and compared.^1^, ^2^, ^3^, ^4^, ^5^, ^6^, ^7^, ^8^, ^9^ Studies have shown that different methods may lead to substantially different effect estimates,^2^, ^3^, ^4^, ^5^, ^8^, ^9^, ^10^ and the optimal method depends on (a) the research aim and (b) whether the medication effect is on the exposure, outcome or a confounder.^10^ If the method used for handling medication effect does not match the research question, substantial bias can be introduced and the interpretation of results will be unclear.^11^

Thus, it is essential to carefully think about the research question when some individuals in a study population use medication that affects the variables in the dataset. In some situations, the research interest could be in the observed measurements, regardless of whether some individuals' measurements are lowered due to antihypertensive medication use; for instance, when the effect of current blood pressure on the course of the disease for patients infected with Covid‐19 is considered. In other cases, blood pressure values that would have been observed if the medication was not administered (sometimes referred to as underlying values^2^, ^12^) could be the primary interest, for example, if the effect of genetic factors on blood pressure are examined. In this instance, a method to correct for the medication effect should be used.

Handling of medication use in epidemiological research has received attention, although this was mainly in methodological papers.^1^, ^2^, ^3^, ^4^, ^5^, ^6^, ^7^, ^8^, ^9^, ^13^, ^14^ There are studies that adopted some of the methods suggested.^15^, ^16^ However, a majority seems to overlook the potential bias due to inadequately handling medication use.^4^ To our knowledge, there has been no systematic review on how medication use is being handled in research practice. Therefore, In this literature review, we aim to investigate which methods are used in observational studies to handle measurements affected by medication, assess how often methods used correspond to the research aims stated in these studies and evaluate the validity of the methods used.

## 2 METHODS

### 2.1 Search strategy

Our search aimed to identify observational studies that included measurements that have been affected by medication use. The search covered three different journal fields; cardiology, diabetes and epidemiology, thereby focusing on blood pressure, glucose or lipid measurements. For each journal field, five journals with the highest impact factors were selected. Table 1 lists the selected journals.

**TABLE 1:** List of selected journals and the number of articles returned from the PubMed search

Cardiology journals (*n =* 258)	Diabetes journals (*n =* 331)	Epidemiology journals (*n =* 688)
*Cardiovascular Research* (4) *Circulation Research* (7) *Circulation* (89) *European Heart Journal* (39) *Hypertension* (119)	*Diabetes* (25) *Diabetes Care* (169) *Diabetes, Obesity & Metabolism* (35) *Diabetologia* (84) *The Lancet Diabetes & Endocrinology* (18)	*American Journal of Epidemiology* (212) *Epidemiology* (108) *European Journal of Epidemiology* (62) *International Journal of Epidemiology* (228) *Journal of Clinical Epidemiology* (78)

To select the publications, we searched PubMed for studies published in the 15 selected journals between January 1, 2015 and December 31, 2019 that used logistic or linear regression. The full search strategies for this step can be found in Supplementary material S1.

The full‐text of the identified papers was screened, and papers that met following inclusion criteria were selected for review: (a) observational studies in adults, (b) sample size larger than 100, (c) aimed to answer etiological questions, (d) performed linear or logistic regression (including linear mixed modeling), and (e) inclusion of any of the following variables: blood pressure‐related measurements (e.g., systolic or diastolic blood pressure, pulse wave velocity), glucose‐related measurements (e.g., glucose level, insulin level, HbA1c, HOMA index) and lipid levels (e.g., cholesterol measures, triglycerides). For studies on type 1 diabetes patients, glucose measurements were not considered because there is no variation in glucose medication use in these patients as insulin treatment is mandatory and unavoidable. If blood pressure related measurements or lipid measurements were used, these studies could be included.

**FIGURE 1:**
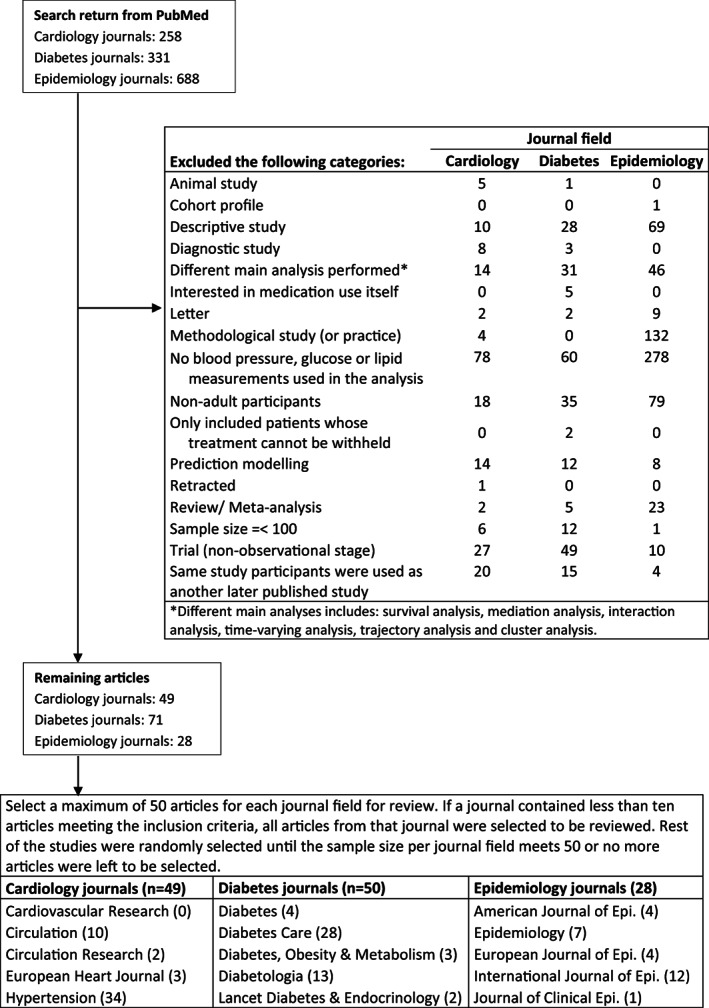
Flow chart of the literature search and screening process

Among the studies that met the inclusion criteria, we selected a maximum of 50 articles to be reviewed from each field. If a specific journal (five per field) contained less than 10 articles meeting the inclusion criteria, all articles from that journal were selected to be reviewed. The rest of the studies were randomly selected until the sample size per journal field met 50 or no more articles were left to be selected. If two or more studies used the same study population within a field, the latest publication was considered (Figure 1).

### 2.2 Data extraction

Data extraction for all 127 papers was independently performed by two reviewers, JC (a PhD candidate in clinical epidemiology) and SlC (a senior statistician and epidemiologist). Disagreements between the two reviewers were resolved during a consensus meeting involving the third reviewer, OMD (a senior epidemiologist and endocrinologist). For each paper, the following general information was extracted:

1. Authors, journal, name of the study/cohort/database.
2. Study population and sample size.
3. Research question with exposure(s) and outcome of interest.
4. Whether linear, logistic regression or both were performed.

For information related to medication use, we extracted the following:

5. Measurements that may have been affected by medication use (blood pressure, glucose, and/or lipid). “Medication use” was defined as the use of drugs that aim to lower blood pressure, glucose, or lipid level.
6. Whether the measurement potentially affected by medication was an exposure, an outcome or a confounder. We used the following rules:
  - When the measurement was mentioned as an “independent variable” and the effect of the variable on the outcome was specifically discussed in the paper, it was coded as an exposure.
  - In Mendelian randomization studies, the exposures in the research questions are the outcomes in the corresponding regression analyses. In this case, we coded the variable as an outcome.
7. Percentage of individuals using medication.
8. Whether details on medication information were given (e.g., type and dose of medication, duration of use).
9. Methods used for handling medication use for each affected variable.

- If different variables had the same role and were handled by the same method, the method was recorded once (e.g., if a study had blood pressure and glucose level as confounding variables and medication use for the both variables are handled by a restriction method, the method was recorded once).
- When multiple models were used to evaluate the same relationship, the most complex model was considered (e.g., when both unadjusted and adjusted analyses were performed to estimate the relationship between the same variables, the adjusted analysis was considered).

c. Justification for the chosen method.
d. Sensitivity analyses for handling medication use.

### 2.3 Assessment of research aims and the validity of the methods used

**FIGURE 2:**
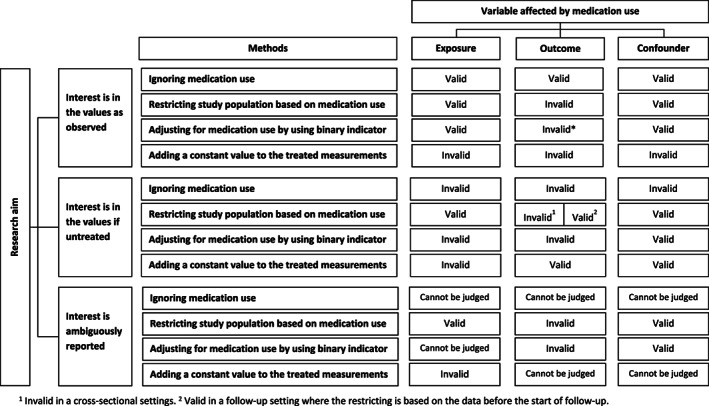
A flow chart for an assessment of valid and invalid approaches for handling medication use. Details on the assessment of the validity of methods used can be found in Appendix S2

We evaluated the validity of methods used for handling medication use based on the research aims of the study and which variable was affected by medication use. Figure 2 displays our evaluation process. In detail, the following steps were taken.

#### 2.3.1 Step I

For each variable affected by medication use, we first evaluated the research aim as stated by the authors, which was categorized as follows:

1. The interest is in the observed values as they are.
2. The interest is in the values that would be observed if no medication was administered (we refer to this as “values if untreated” or “untreated values” in the further text).
3. The interest is ambiguously reported.

#### 2.3.2 Step II

The validity of the method used for each variable was evaluated in relationship to the research aim and whether the affected variable is an exposure, an outcome, or a confounder. The assessment on whether the methods used are in general valid or invalid was based on recommendations from previous methodological studies.^2^, ^3^, ^4^, ^5^, ^6^, ^10^, ^11^, ^17^, ^18^, ^19^, ^20^, ^21^, ^22^, ^23^, ^24^, ^25^, ^26^ For example, restricting the study population to non‐medication users was considered valid when the exposure or a confounder is affected by medication use regardless of whether the research aim is in the values as observed or if untreated. This is because the restriction on a proxy variable of the exposure or a confounder (medication use, in this case) in general would lead to a selection of a subgroup without introducing selection bias.^21^ Contrarily, the restriction method was considered invalid regardless of the research aim when the outcome is affected by medication use. Selection on medication use, an event occurred after the follow‐up started and related to the outcome, would introduce selection bias.^2^, ^21^, ^22^, ^24^, ^25^, ^26^ A complete discussion of all possible options can be found in Supplementary material S2.

## 3 RESULTS

Our search strategy in PubMed retrieved 258 articles in cardiology journals, 331 articles in diabetes journals and 688 articles in epidemiology journals (see Table 1 for the number of papers and Figure 1 for flow‐chart). After the screening process, 49 articles in the cardiology field, 73 articles in the diabetes field, and 28 articles in the epidemiology field remained. For the diabetes field, a subset of 50 articles was selected as described in the methods section. We included 49 articles from cardiology journals, 50 articles from diabetes journals, and 28 articles from epidemiology journals, for a total of 127 studies.

### 3.1 Summaries of reviewed articles

Supplementary material S3 displays the complete list of the reviewed articles and extracted information of each article. Table 2 provides a summary of the included studies. Overall, the measurement affected by medication use was most often a confounding variable (In 56% of the studies), followed by an outcome (42%) and/or an exposure (35%). In the epidemiology journals, affected outcomes were more often present (64%). Sample sizes varied largely between the reviewed articles and were generally larger in the epidemiology journals. Included studies performed linear regression analysis (59%), logistic regression analysis (40%), and/or linear mixed modeling (9%).

**TABLE 2:** Summaries of reviewed articles

	Journal field			
	All journals (*n =* 127)	Cardiology (*n =* 49)	Diabetes (*n =* 50)	Epidemiology (*n =* 28)
Affected variables in the analysis^a^, *n* (%)				
Exposure	45 (35.4)	21 (42.9)	20 (40.0)	4 (14.3)
Outcome	53 (41.7)	17 (34.7)	18 (36.0)	18 (64.3)
Confounder	71 (55.9)	29 (59.2)	33 (66.0)	9 (32.1)
Sample size (median [min, max])	1540	1746	1147	2514
	[122, 615 035]	[122, 615 035]	[122, 222 773]	[277, 486 936]
Type of analysis^a^, *n* [%]				
Linear regression	75 (59.1)	29 (59.2)	26 (52.0)	20 (71.4)
Logistic regression	51 (40.2)	19 (38.8)	25 (50.0)	7 (25.0)
(Generalized) Linear mixed model	12 (9.4)	6 (12.2)	4 (8.0)	2 (7.1)
Percentage of medication use				
Reported or traceable for all variables	49 (38.6)	24 (49.0)	19 (38.0)	6 (21.4)
Reported for some variables	18 (14.2)	6 (12.2)	9 (18.0)	3 (10.7)
Not reported	60 (47.2)	19 (38.8)	22 (44.0)	19 (67.9)
Medication users percentage among the reported (median [min, max])	32.0 [0, 100]	22.0 [0, 91]	54.6 [0, 100]	11.7 [1.3, 59]
Details of medication information reported	9 (7.1)	6 (12.2)	2 (4.0)	1 (3.6)

^a^ Exceed 100% when added up, because more than one analysis was performed in some studies.

Overall, a majority of the studies did not report the percentage of medication users (47%) or only reported medication use for part of the variables affected (14%). Among the studies which fully or partially provided information on the percentage of medication users, the median percentage of medication users was 32%. The percentage of medication users ranged from 0 to 100, because some studies restricted their study population to medication users or non‐users. Details of medication use, such as dose or prescription frequency, were seldom given (7%).

### 3.2 Methods used for handling medication use

Table 3 summarizes the methods used for handling measurements affected by medication use. Lists of the studies using each method can be found in Supplementary material S4. A large number of studies did not use any method specifically for handling medication use (58% when medication use was in the exposure, 53% when in the outcome, and 45% when in a confounder). Restricting the analysis to a certain subpopulation was frequently used (for exposure: 22%, outcome: 23% and confounder: 10%). Some studies restricted their study population to medication users or non‐medication users. Others restricted the analyses to subgroups that were partly defined based on medication use; such as individuals without hypertension, defined as people not using antihypertensive drug and having normal blood pressure levels.

**TABLE 3:** Frequency of methods used for handling medication use in main analyses, *n* (%)

Affected measurement	Methods	Journal field			
		All journals	Cardiology	Diabetes	Epidemiology
Exposure	Ignoring medication use	26 (57.8)	13 (61.9)	9 (45.0)	4 (100)
	Restricting study population	10 (22.2)	4 (19)	6 (30.0)	—
	To medication users	1	—	1	—
	To non‐medication users	3	2	1	—
	To non‐medication users and having normal values	6	2	4	—
	Adjusting as a binary covariate	8 (17.8)	3 (14.3)	5 (25.0)	—
	Using medication (yes/no)	7	2	5	—
	Using medication or having high values (yes/no)	1	1	—	—
	Adding a constant value to the treated measurements	1 (2.2)	1 (4.8)	—	—
	Subtotal	45 (100)	21 (100)	20 (100)	4 (100)
Outcome	Ignoring medication use	28 (52.8)	9 (52.9)	8 (44.4)	11 (61.1)
	Restricting study population	12 (22.6)	1 (5.9)	7 (38.9)	4 (22.2)
	To non‐medication users	7	1	3	3
	To non‐medication users and having normal clinical values	5	—	4	1
	Adjusting as a binary covariate	10 (18.9)	5 (29.4)	3 (16.7)	2 (11.1)
	Using medication (yes/no)	10	5	3	2
	Adding a constant value to the treated measurements	3 (5.7)	2 (11.8)	—	1 (5.6)
	Subtotal	53 (100)	17 (100)	18 (100)	18 (100)
Confounder	Ignoring medication use	32 (45.1)	9 (31.0)	17 (51.5)	6 (66.7)
	Restricting study population	7 (9.9)	2 (6.9)	5 (15.2)	—
	To medication users	3	—	3	—
	To non‐medication users	1	—	1	—
	To non‐medication users and having normal clinical values	3	2 (6.9)	1	—
	Adjusting as a binary covariate	32 (45.1)	18 (62.1)	11 (33.3)	3 (33.3)
	Using medication (yes/no)	26	13	10	3
	Using medication or having high values (yes/no)	6	5	1	
	Subtotal	71 (100)	31 (100)	33 (100)	9 (100)
Justification for the chosen method given		12 (9.4)	6 (12.2)	3 (6.0)	3 (10.7)
Sensitivity analysis performed		22 (17.3)	9 (18.4)	8 (16.0)	5 (17.9)

*Note*: The sum of the subtotals exceed the total number of articles included for each journals field (49 for cardiology journals, 50 for diabetes journals, and 28 for epidemiology journals) because more than one variable was affected by medication use in some studies.

A binary covariate in a regression model was the next most used method for exposures (18%) and outcomes (19%). For confounders, it was one of the most used methods (45%). The binary variable used for the adjustment was often “using medication (yes/no).” However, one study adjusted for “using medication or having high value (yes/no)” (e.g., hypertension vs. no hypertension, while defining hypertension as taking antihypertensive drugs *or* having blood pressure above a certain level).

Adding an estimate of the mean medication effect to treated values was adopted only in four studies. One study used this method for handling medication use in the exposure. No study used any of the more advanced methods suggested in the literature, such as quantile regression,^3^ censored normal regression^2^ or Heckman's treatment model.^4^, ^5^

In total, only 10 studies (8%) explicitly provided justification for the chosen methods for handling medication use. Given justifications, however, may not reflect the validity of the methods used. Sensitivity analyses were performed in 21 studies (16%) in total. A list of methods used in the sensitivity analyses can be found in Supplementary material S5.

### 3.3 Assessment of research aim‐analysis match and validity of the methods used

The results of the assessment of the methods used for handling medication use are summarized in Table 4. In a majority of the studies, it was unclear if whether the research interest was in the values as observed or in untreated values. Thus, the validity of the used methods often could not be judged properly (exposure: 56%, outcome: 36%, confounder: 45%). Overall, no noticeable difference in performance was observed across the journal fields.

**TABLE 4:** Assessment of the research question‐analysis match and the validity of used methods, *n* (%). The percentage add up to 100 per affected variable

Affected variable	Research aim	Validity	Journal field			
			All journals (*n =* 127)	Cardiology (*n =* 49)	Diabetes (*n =* 50)	Epidemiology (*n =* 28)
Exposure	As observed	Valid	6 (13.3)	1 (4.8)	5 (25.0)	—
		Invalid	—	—	—	—
	If untreated	Valid	6 (13.3)	3 (14.3)	3 (15.0)	—
		Invalid	5 (11.1)	3 (14.3)	1 (5.0)	1 (25.0)
	Ambiguously reported	Valid	3 (6.7)	1 (4.8)	2 (10.0)	—
		Invalid	—	—	—	—
		Cannot be judged	25 (55.6)	13 (61.9)	9 (45.0)	3 (75.0)
Outcome	As observed	Valid	2 (3.8)	—	2 (11.1)	—
		Invalid	1 (1.9)	—	1 (5.6)	—
	If untreated	Valid	2 (3.8)	1 (5.9)	—	1 (5.6)
		Invalid	19 (35.8)	4 (23.5)	10 (55.6)	5 (27.8)
	Ambiguously reported	Valid	—	—	—	—
		Invalid	11 (20.8)	4 (23.5)	2 (11.1)	5 (27.8)
		Cannot be judged	18 (34.0)	8 (47.1)	3 (16.7)	7 (38.9)
Confounder	As observed	Valid	4 (5.6)	1 (3.4)	3 (9.1)	—
		Invalid	—	—	—	—
	If untreated	Valid	4 (5.6)	3 (10.3)	1 (3.0)	—
		Invalid	—	—	—	—
	Ambiguously reported	Valid	31 (43.7)	16 (55.2)	12 (36.4)	3 (33.3)
		Invalid	—	—	—	—
		Cannot be judged	32 (45.1)	9 (31.0)	17 (51.5)	6 (66.7)

*Note*: The percentages add up to 100 per “affected variable.”

In all studies where the interest explicitly was in observed exposure values, medication use was also ignored in the analyses. When interest was in untreated exposure values (11 analyses), most often the analysis was restricted to untreated individuals, which is considered in general a valid approach. However, in 5/11 analyses, invalid approaches were used; such as, ignoring the treatment, adjusting for medication use as binary covariates or adding a constant value. In 3/28 analyses where the research aim for the exposure variable was ambiguous, the study population was restricted to untreated individuals, which we considered a valid approach for all research aims.

When the outcome was an affected variable, we found only three out of 53 analyses that were undoubtedly interested in the values as observed. Among these, two analyses ignored medication use accordingly. However, one used a valid method which is adjusting for medication use as a binary covariate. More often, the studies were found to be interested in the outcome values if untreated. However, in most cases (19/21 analyses), invalid approaches, such as restricting the study population or adjusting using a binary covariate, were used. When the research aim for the outcome variable was ambiguous, the affected outcome was often handled with methods that are prone to yield biased causal effect regardless of the research aim; for example, as restricting the study population in a cross‐sectional setting or adjusting using a binary covariate.

For confounders affected, only in eight out of 71 cases it was clear whether interest was in observed values (*n =* 4) or in untreated values (*n =* 4). Valid methods were used in these cases. When the aim was unclear, often (31/63) medication use was added as an additional covariate to the regression model. This approach is considered valid both when interest is in observed values, (where medication use could be an extra confounder), and also when interest is in unaffected values (in which case adding both medication use and the observed value will account for most of the confounding of the underlying unaffected values).

## 4 DISCUSSION

In this review, we empirically assessed how variables affected by medication use are handled in observational etiological studies. Our review showed that a large proportion of the studies did not provide clear research aims stating whether their interest was in the observed or the untreated underlying values and methods in general considered invalid, such as restricting the study population to non‐medication users when the outcome is affected by medication use, were often used. Notably, a justification for the chosen method was rarely given, and the number of medication users was not reported or insufficiently reported in more than a half of the studies. These findings suggest that there is low awareness of potential bias by medication use.

The median percentage of medication users in our review was 31%, in which case the estimated effect may differ considerably depending on whether the interest is in the observed values or the underlying unaffected values. Even when the number of medication use is low, differences can still be substantial if the effect of medication is large. More information on the direction and magnitude of bias when interest is in the underlying unaffected values can be found in several methodological studies.^2^, ^3^, ^10^ Factors that may play a role include, but are not limited to, different types of medication and doses, heterogeneity of medication effect across the individuals, medication effect being canceled/ enhanced by other interventions or time‐varying aspect of medication use. Such information heavily relies on content knowledge. Thus, we urge clinical researchers to provide and discuss relevant information on medication used in their study population.

We found that invalid methods were especially prevalent when the affected variable was the outcome. Often the analysis was performed conditional on medication use. Although the bias due to selection on events related to the outcome has been discussed extensively in the literature,^2^, ^21^, ^22^, ^24^, ^25^, ^26^ it seemed that such consideration was often not taken into account. We also observed that the research aim was most often ambiguously reported for confounding variables affected by medication use. This is not surprising since confounders are mostly not the variables of main interests. However, inadequately handling medication use in confounding variables can lead to bias.^10^

We noticed that recommendations in methodological papers were seldom applied. For example, Tobin et al.^2^ recommended adding a constant value to measurements of treated values of an outcome variable when interest is in the underlying unaffected values and stressed the necessity of sensitivity analysis to determine the robustness to the particular choice of constant. In our review, none of the four studies which applied this method tested the robustness of their choice of constant. Additionally, no study was found to use any of the more advanced statistical methods previously suggested.^2^, ^3^, ^5^, ^10^ This may call for methodological papers in clinical journals that provide practical guidelines and tutorials on when and how to apply corrections for medication use in applied clinical research.

We only included studies that used linear regression, logistic regression and mixed linear models. However, potential bias due to measurements affected by medication use is present in any study where a mixed study population of medication users and non‐users exists.^14^ In complex settings, such as when medication use is an effect modifier or a mediator or when there is time‐varying medication use, extra caution would be needed.^1^, ^13^ Handling of medication use also plays a role when continuous variables are being categorized. For example, when categorizing glucose values in high versus normal, the distinction could be made based on untreated values, where patients on medication are classified as high glucose even if their glucose levels are regulated. These approaches would be considered valid once medication users are classified correctly; however, the power may be lower.^3^, ^27^, ^28^

## 5 CONCLUSION

Our review has shown that potential bias due to medication use is often overlooked and that decisions on handling medication use are frequently made without valid justification. We urge researchers to provide clear information on medication use, consciously decide on a method for handling medication use based on their research question and communicate the rationale behind their decision.

## CONFLICT OF INTEREST

The authors declare no conflicts of interest.

## Supporting information


**Appendix S1**: Supporting information.

**Appendix S2**: Supporting information.

**Appendix S3**: Supporting information.

**Appendix S4**: Supporting information.

**Appendix S5**: Supporting information.

## Data Availability

No new data were generated or analyzed in support of this research.
