# Supplementary material for: How measurements affected by medication use are reported and handled in observational research: A literature review
Source: Pharmacoepidemiol Drug Saf. 2022 May 4;31(7):739–48. doi: 10.1002/pds.5437 (PMC9321697; doi:10.1002/pds.5437)
Supplement: Supplementary file 1 — Appendix S1: Supporting information. [file PDS-31-739-s002.docx]

**Supplementary material 1. PubMed search strategy**

PubMed: <http://www.ncbi.nlm.nih.gov/pubmed?otool=leiden>

(("Hypertension"[Journal] OR "European Heart Journal"[Journal] OR "Circulation"[Journal] OR "Circulation research"[Journal] OR "Cardiovascular research"[Journal]) AND ("linear regression"[tw] OR "linear regressions"[tw] OR linear regres*[tw] OR "logistic regression"[tw] OR "logistic regressions"[tw] OR logistic regres*[tw] OR "Linear Models"[Mesh] OR "Linear Model"[tw] OR "Linear Models"[tw] OR "Logistic Models"[Mesh] OR "Logistic Model"[tw] OR "Logistic Models"[tw]) AND ("2015/01/01"[PDat] : "2019/12/31"[PDat]))

(("The lancet. Diabetes & endocrinology"[Journal] OR "Diabetes care"[Journal] OR "Diabetes. obesity & metabolism"[Journal] OR "Diabetes"[Journal] OR "Diabetologia"[Journal]) AND ("linear regression"[tw] OR "linear regressions"[tw] OR linear regres*[tw] OR "logistic regression"[tw] OR "logistic regressions"[tw] OR logistic regres*[tw] OR "Linear Models"[Mesh] OR "Linear Model"[tw] OR "Linear Models"[tw] OR "Logistic Models"[Mesh] OR "Logistic Model"[tw] OR "Logistic Models"[tw]) AND ("2015/01/01"[PDat] : "2019/12/31"[PDat]))

(("European journal of epidemiology"[Jour] OR "International journal of epidemiology"[Jour] OR "Epidemiology"[Jour] OR "American journal of epidemiology"[Jour] OR "journal of clinical epidemiology"[Jour]) AND ("linear regression"[tw] OR "linear regressions"[tw] OR linear regres*[tw] OR "logistic regression"[tw] OR "logistic regressions"[tw] OR logistic regres*[tw] OR "Linear Models"[Mesh] OR "Linear Model"[tw] OR "Linear Models"[tw] OR "Logistic Models"[Mesh] OR "Logistic Model"[tw] OR "Logistic Models"[tw]) AND ("2015/01/01"[PDat] : "2019/12/31"[PDat]))
