# Supplementary material for: How measurements affected by medication use are reported and handled in observational research: A literature review
Source: Pharmacoepidemiol Drug Saf. 2022 May 4;31(7):739–48. doi: 10.1002/pds.5437 (PMC9321697; doi:10.1002/pds.5437)
Supplement: Supplementary file 2 — Appendix S2: Supporting information. [file PDS-31-739-s001.docx]

**Supplementary material 2. Details on the assessment of the validity of used methods.**

1. When the affected variable is an exposure
   1. When the interest is in the values as observed
      1. Ignoring medication use is valid.
      2. Restricting the study population based on medication use is considered valid. The method can yield unbiased estimates in the selected subpopulation, given that variables affecting both medication use and the outcome are correctly adjusted (1, 2). Results cannot be extrapolated to the excluded population when effect heterogeneity is present.
      3. Adjusting for medication use as a binary covariate is considered valid, as the medication use occurs prior to the exposure variable is measured. Adjusting for medication use may be needed if it also affects the outcome, in which case medication use is an confounder (3).
      4. Adding a constant value (the estimated mean medication effect) to the treated measurements) is considered invalid. This is because the method does not account for the variability in medication effect between medication users (2, 4).
   2. When the interest is in the values if untreated
      1. Ignoring medication use is invalid.
      2. Restricting the study population based on medication use is considered valid. The method will yield a valid estimate of the effect of the exposure on the outcome in the subpopulation, under the same considerations as 1.1.2.
      3. Adjusting for medication use as a binary covariate is considered invalid. The effect of medication is in general not the same in all individuals. Therefore, applying this method will likely lead to an underestimation of the association between the exposure and the outcome, due to the fact that the medication effect on the exposure level cannot be completely account for (2, 4).
      4. Adding a constant value to the treatment is considered invalid, because the method does not account for the variability in medication effect between medication users. This phenomenon is described in literature on measurement error (see reference) (2, 4).
   3. When the interest is ambiguous
      1. The validity of ignoring medication use cannot be judged.
      2. Restricting the study population based on medication use is considered valid, because it is a valid approach in either cases where the research aim is in the values as observed or the values if untreated.
      3. The validity of adjustment for a binary indicator cannot be judged.
      4. Adding a constant value to the treated measurements is invalid.
2. When the affected variable is an outcome
   1. When the interest is in the values as observed
      1. Ignoring medication use is valid.
      2. Restricting the study population based on medication use is invalid, because of selection on intercurrent events may lead to selection bias (1, 5, 6).
      3. Adjusting for medication use with a binary indicator is considered invalid. The method may lead to selection bias (collider bias) due to indirect conditioning on intercurrent events and the outcome variable (1, 5, 6).
      4. Adding a constant value is invalid.
   2. When the interest is in the values, if untreated
      1. Ignoring medication use is invalid.
      2. Restricting the study population based on medication use is invalid as the method introduces selection bias. (1, 7-9)
      3. Adjusting for medication use is invalid as it introduces collider bias (5, 8).
      4. Adding a constant value to the treated measurement is a valid approach (2, 8, 10, 11).
   3. When the interest is ambiguous
      1. The validity of ignoring medication use cannot be judged.
      2. Restricting the study population based on medication use is invalid, because it is invalid for both when interest was in the values as observed or the values if untreated values.
      3. Adjusting for medication use is invalid when interest was in observed and in untreated values.
      4. The validity of adding a constant value to the treated measurement cannot be judged.
3. When the affected variable is a confounder
   1. When the interest is in the values as observed
      1. Ignoring medication use is valid.
      2. Restricting the study population based on medication use is considered valid under the same considerations as 1.1.2. The method serves as accounting for confounding by restriction (5).
      3. Adjusting for medication use is valid. The method is comparable to adjusting for a proxy confounder (5, 12).
      4. Adding a constant value to treated measurements is considered invalid.
   2. When the interest is in the values, if untreated
      1. Ignoring medication use is invalid.
      2. Restricting the study population based on medication use is valid for the same reason as 3.1.2.
      3. Adjusting for medication use is valid for the same reason as 3.1.3.
      4. Adding a constant value to the treatment measurement is considered valid because simulation showed that this approach would handle most of the confounding (2).
   3. When the interest is ambiguous
      1. The validity of ignoring medication use cannot be judged.
      2. Restricting the study population based on medication use is valid for the same reason as 1.1.2.
      3. Adjusting for medication use is valid for the same reason as 3.1.3.
      4. The validity of adding a constant value to the treated measurement cannot be judged.

More advanced methods are available (not shown in Figure 2) when the study interest is in the underlying value that is *not* affected by medication use. For instance, censored normal regression (8), quantile regression (10) and Heckman’s treatment model (13), could be used under certain assumptions when the outcome is affected. Methods for correcting differential measurement error (e.g., regression calibration with adding mean treatment effect) could be used (4, 14) for an exposure affected by medication use. However, a judgment about the validity of these methods cannot be made if the study aim is ambiguous.

1. Hernán M, S H-D, Robins J. A Structural Approach to Selection Bias. *Epidemiology* 2004;15(5):615-25.

2. Choi J, Dekkers OM, le Cessie S. A comparison of different methods for handling measurements affected by medication use. *(ready to submit)*.

3. Rubin DB. For objective causal inference, design trumps analysis. *The Annals of Applied Statistics* 2008;2(3):808-40, 33.

4. Carroll RJ, Ruppert D, Stefanski LA, et al. *Measurement error in nonlinear models: a modern perspective*. Chapman and Hall/CRC; 2006.

5. Hernán M, Robins J. *Causal inference: What if*. Boca Raton: Chapman & Hall/CRC; 2020.

6. Cole SR, Platt RW, Schisterman EF, et al. Illustrating bias due to conditioning on a collider. *International Journal of Epidemiology* 2009;39(2):417-20.

7. Hernán M. Invited Commentary: Selection Bias Without Colliders. *American Journal of Epidemiology* 2017;185(11):1048-50.

8. Tobin MD, Sheehan NA, Scurrah KJ, et al. Adjusting for treatment effects in studies of quantitative traits: antihypertensive therapy and systolic blood pressure. *Statistics in Medicine* 2005;24(19):2911-35.

9. Elwert F, Winship C. Endogenous Selection Bias: The Problem of Conditioning on a Collider Variable. *Annu Rev Sociol* 2014;40:31-53.

10. White IR, Koupilova I, Carpenter J. The use of regression models for medians when observed outcomes may be modified by interventions. 2003;22(7):1083-96.

11. Tanamas SK, Hanson RL, Nelson RG, et al. Effect of different methods of accounting for antihypertensive treatment when assessing the relationship between diabetes or obesity and systolic blood pressure. *Journal of Diabetes and its Complications* 2017;31(4):693-9.

12. VanderWeele TJ. Principles of confounder selection. *European Journal of Epidemiology* 2019;34(3):211-9.

13. Spieker AJ, Delaney JAC, McClelland RL. Evaluating the treatment effects model for estimation of cross-sectional associations between risk factors and cardiovascular biomarkers influenced by medication use. *Pharmacoepidemiology and drug safety* 2015;24(12):1286-96.

14. Hutcheon JA, Chiolero A, Hanley JA. Random measurement error and regression dilution bias. *BMJ* 2010;340:c2289.
