# Supplementary material for: How measurements affected by medication use are reported and handled in observational research: A literature review
Source: Pharmacoepidemiol Drug Saf. 2022 May 4;31(7):739–48. doi: 10.1002/pds.5437 (PMC9321697; doi:10.1002/pds.5437)
Supplement: Supplementary file 3 — Appendix S3: Supporting information. [file PDS-31-739-s004.docx]

**Supplementary material 3. Studies identified to be using each method for handling variables affected by medication use**

| **Affected measurement** |  | **Journal field** | | |
| --- | --- | --- | --- | --- |
|  |  | **Cardiology** | **Diabetes** | **Epidemiology** |
| **Exposure** | **Ignoring medication use** | (1-11) | (12-19) | (20-23) |
|  | **Restricting the study population** |  |  | - |
|  | to medication users | - | (24) | - |
|  | to non-medication users | (25, 26) | (27) | - |
|  | to individuals free of a condition | (25, 28) | (13, 29-31) | - |
|  | **Adjusting as a binary covariate** |  |  | - |
|  | using medication (yes/no) | (28, 32) | (33-37) | - |
|  | having a condition (yes/no) | (38) | - |  |
|  | **Adding a constant value to the treated measurements** | (39) | - | - |
| **Outcome** | **Ignoring medication use** | (2, 4, 6, 40-45) | (15, 46-52) | (53-63) |
|  | **Restricting study population** |  |  |  |
|  | to non-medication users | (64) | (17, 65, 66) | (67-69) |
|  | to individuals free of a condition | - | (15, 29, 70, 71) | (72) |
|  | **Adjusting as a binary covariate** |  |  |  |
|  | using medication (yes/no) | (73-77) | (33, 78, 79) | (72, 80) |
|  | **Adding a constant value to the treated measurements** | (81, 82) | - | (83) |
| **Confounder** | **Ignoring medication use** | (5, 76, 84-89) | (27, 31, 37, 47, 70, 90-101) | (58, 102-106) |
|  | **Restricting study population** |  |  |  |
|  | to medication users | - | (93, 107, 108) | - |
|  | to non-medication users | - | (99) | - |
|  | to individuals free of a condition | (88, 109) | (52) | - |
|  | **Adjusting as a binary covariate** |  |  |  |
|  | using medication (yes/no) | (32, 74, 76, 77, 87, 89, 109-115) | (17, 19, 70, 92, 99, 107, 116-119) | (104, 120, 121) |
|  | having a condition (yes/no) | (122-126) | (78) | - |
| **Justification for the chosen method given** | | (26, 39, 64, 81, 82, 125) | (17, 66, 117) | (23, 72, 83) |
| **Sensitivity analysis performed** | | (4, 5, 10, 32, 41, 45, 76, 81, 84) | (15, 19, 34, 47-49, 51, 127) | (57, 59, 69, 80, 83) |

1. Gransbo K, Almgren P, Nilsson PM, et al. Risk factor exposure in individuals free from cardiovascular disease differs according to age at first myocardial infarction. *European heart journal* 2016;37(25):1977-81.

2. Ranque B, Menet A, Boutouyrie P, et al. Arterial Stiffness Impairment in Sickle Cell Disease Associated With Chronic Vascular Complications: The Multinational African CADRE Study. *Circulation* 2016;134(13):923-33.

3. Catena C, Colussi G, Verheyen ND, et al. Moderate Alcohol Consumption Is Associated With Left Ventricular Diastolic Dysfunction in Nonalcoholic Hypertensive Patients. *Hypertension (Dallas, Tex : 1979)* 2016;68(5):1208-16.

4. Koivistoinen T, Lyytikainen LP, Aatola H, et al. Pulse Wave Velocity Predicts the Progression of Blood Pressure and Development of Hypertension in Young Adults. *Hypertension (Dallas, Tex : 1979)* 2018;71(3):451-6.

5. Mahinrad S, Kurian S, Garner CR, et al. Cumulative Blood Pressure Exposure During Young Adulthood and Mobility and Cognitive Function in Midlife. *Circulation* 2020;141(9):712-24.

6. Naidoo S, Kagura J, Fabian J, et al. Early Life Factors and Longitudinal Blood Pressure Trajectories Are Associated With Elevated Blood Pressure in Early Adulthood. *Hypertension (Dallas, Tex : 1979)* 2019;73(2):301-9.

7. Birukov A, Andersen LB, Herse F, et al. Aldosterone, Salt, and Potassium Intakes as Predictors of Pregnancy Outcome, Including Preeclampsia. *Hypertension (Dallas, Tex : 1979)* 2019;74(2):391-8.

8. Hung CS, Sung SH, Liao CW, et al. Aldosterone Induces Vascular Damage. *Hypertension (Dallas, Tex : 1979)* 2019;74(3):623-9.

9. Martinez-Martinez E, Lopez-Andres N, Jurado-Lopez R, et al. Galectin-3 Participates in Cardiovascular Remodeling Associated With Obesity. *Hypertension (Dallas, Tex : 1979)* 2015;66(5):961-9.

10. Glodzik L, Rusinek H, Tsui W, et al. Different Relationship Between Systolic Blood Pressure and Cerebral Perfusion in Subjects With and Without Hypertension. *Hypertension (Dallas, Tex : 1979)* 2019;73(1):197-205.

11. Toledo C, Thomas G, Schold JD, et al. Renal resistive index and mortality in chronic kidney disease. *Hypertension (Dallas, Tex : 1979)* 2015;66(2):382-8.

12. Akehi Y, Yanase T, Motonaga R, et al. High Prevalence of Diabetes in Patients With Primary Aldosteronism (PA) Associated With Subclinical Hypercortisolism and Prediabetes More Prevalent in Bilateral Than Unilateral PA: A Large, Multicenter Cohort Study in Japan. *Diabetes care* 2019;42(5):938-45.

13. Szili-Torok T, Annema W, Anderson JLC, et al. HDL Cholesterol Efflux Predicts Incident New-Onset Diabetes After Transplantation (NODAT) in Renal Transplant Recipients Independent of HDL Cholesterol Levels. *Diabetes* 2019;68(10):1915-23.

14. Neergaard JS, Dragsbaek K, Christiansen C, et al. Metabolic Syndrome, Insulin Resistance, and Cognitive Dysfunction: Does Your Metabolic Profile Affect Your Brain? *Diabetes* 2017;66(7):1957-63.

15. Bonnefond A, Yengo L, Le May C, et al. The loss-of-function PCSK9 p.R46L genetic variant does not alter glucose homeostasis. *Diabetologia* 2015;58(9):2051-5.

16. Feinkohl I, Keller M, Robertson CM, et al. Cardiovascular risk factors and cognitive decline in older people with type 2 diabetes. *Diabetologia* 2015;58(7):1637-45.

17. Aroner SA, Furtado JD, Sacks FM, et al. Apolipoprotein C-III and its defined lipoprotein subspecies in relation to incident diabetes: the Multi-Ethnic Study of Atherosclerosis. *Diabetologia* 2019;62(6):981-92.

18. Callaghan BC, Xia R, Banerjee M, et al. Metabolic Syndrome Components Are Associated With Symptomatic Polyneuropathy Independent of Glycemic Status. *Diabetes care* 2016;39(5):801-7.

19. Parrinello CM, Sharrett AR, Maruthur NM, et al. Racial Differences in and Prognostic Value of Biomarkers of Hyperglycemia. *Diabetes Care* 2016;39(4):589-95.

20. Huang CC, Lee JC, Lin KC, et al. Exposure Duration and History of Hypertension Predicted Neurological Sequelae in Patients with Carbon Monoxide Poisoning. *Epidemiology (Cambridge, Mass)* 2019;30 Suppl 1:S76-s81.

21. Xiao Q, Moore SC, Keadle SK, et al. Objectively measured physical activity and plasma metabolomics in the Shanghai Physical Activity Study. *International journal of epidemiology* 2016;45(5):1433-44.

22. Simeon V, Chiodini P, Mattiello A, et al. Dietary glycemic load and risk of cognitive impairment in women: findings from the EPIC-Naples cohort. *European journal of epidemiology* 2015;30(5):425-33.

23. Hofmann JN, Corley DA, Zhao WK, et al. Chronic kidney disease and risk of renal cell carcinoma: differences by race. *Epidemiology (Cambridge, Mass)* 2015;26(1):59-67.

24. Home P, Calvi-Gries F, Blonde L, et al. Clinical correlates of hypoglycaemia over 4 years in people with type 2 diabetes starting insulin: An analysis from the CREDIT study. *Diabetes, obesity & metabolism* 2018;20(4):921-9.

25. Perticone F, Perticone M, Maio R, et al. Serum alkaline phosphatase negatively affects endothelium-dependent vasodilation in naive hypertensive patients. *Hypertension (Dallas, Tex : 1979)* 2015;66(4):874-80.

26. Tedla YG, Yano Y, Carnethon M, et al. Association Between Long-Term Blood Pressure Variability and 10-Year Progression in Arterial Stiffness: The Multiethnic Study of Atherosclerosis. *Hypertension (Dallas, Tex : 1979)* 2017;69(1):118-27.

27. Ekblad LL, Rinne JO, Puukka PJ, et al. Insulin resistance is associated with poorer verbal fluency performance in women. *Diabetologia* 2015;58(11):2545-53.

28. Flores-Guerrero JL, Groothof D, Connelly MA, et al. Concentration of Branched-Chain Amino Acids Is a Strong Risk Marker for Incident Hypertension. *Hypertension (Dallas, Tex : 1979)* 2019;74(6):1428-35.

29. Daniele G, Winnier D, Mari A, et al. Sclerostin and Insulin Resistance in Prediabetes: Evidence of a Cross Talk Between Bone and Glucose Metabolism. *Diabetes care* 2015;38(8):1509-17.

30. Norhammar A, Kjellstrom B, Habib N, et al. Undetected Dysglycemia Is an Important Risk Factor for Two Common Diseases, Myocardial Infarction and Periodontitis: A Report From the PAROKRANK Study. *Diabetes care* 2019;42(8):1504-11.

31. Ruijgrok C, Dekker JM, Beulens JW, et al. Size and shape of the associations of glucose, HbA1c, insulin and HOMA-IR with incident type 2 diabetes: the Hoorn Study. *Diabetologia* 2018;61(1):93-100.

32. Fan F, Qi L, Jia J, et al. Noninvasive Central Systolic Blood Pressure Is More Strongly Related to Kidney Function Decline Than Peripheral Systolic Blood Pressure in a Chinese Community-Based Population. *Hypertension (Dallas, Tex : 1979)* 2016;67(6):1166-72.

33. Lithovius R, Gordin D, Forsblom C, et al. Ambulatory blood pressure and arterial stiffness in individuals with type 1 diabetes. *Diabetologia* 2018;61(9):1935-45.

34. Punthakee Z, Iglesias PP, Alonso-Coello P, et al. Association of preoperative glucose concentration with myocardial injury and death after non-cardiac surgery (GlucoVISION): a prospective cohort study. *The lancet Diabetes & endocrinology* 2018;6(10):790-7.

35. Ferreira MT, Leite NC, Cardoso CR, et al. Correlates of aortic stiffness progression in patients with type 2 diabetes: importance of glycemic control: the Rio de Janeiro type 2 diabetes cohort study. *Diabetes care* 2015;38(5):897-904.

36. Misra-Hebert AD, Pantalone KM, Ji X, et al. Patient Characteristics Associated With Severe Hypoglycemia in a Type 2 Diabetes Cohort in a Large, Integrated Health Care System From 2006 to 2015. *Diabetes care* 2018;41(6):1164-71.

37. Guerrero-Berroa E, Ravona-Springer R, Heymann A, et al. Haptoglobin genotype modulates the relationships of glycaemic control with cognitive function in elderly individuals with type 2 diabetes. *Diabetologia* 2015;58(4):736-44.

38. Hess CN, Wang TY, McCoy LA, et al. Unplanned Inpatient and Observation Rehospitalizations After Acute Myocardial Infarction: Insights From the Treatment With Adenosine Diphosphate Receptor Inhibitors: Longitudinal Assessment of Treatment Patterns and Events After Acute Coronary Syndrome (TRANSLATE-ACS) Study. *Circulation* 2016;133(5):493-501.

39. Huang Y, Ollikainen M, Sipila P, et al. Genetic and Environmental Effects on Gene Expression Signatures of Blood Pressure: A Transcriptome-Wide Twin Study. *Hypertension (Dallas, Tex : 1979)* 2018;71(3):457-64.

40. Hovi P, Vohr B, Ment LR, et al. Blood Pressure in Young Adults Born at Very Low Birth Weight: Adults Born Preterm International Collaboration. *Hypertension (Dallas, Tex : 1979)* 2016;68(4):880-7.

41. Cai Y, Hansell AL, Blangiardo M, et al. Long-term exposure to road traffic noise, ambient air pollution, and cardiovascular risk factors in the HUNT and lifelines cohorts. *European heart journal* 2017;38(29):2290-6.

42. Narayan HK, Finkelman B, French B, et al. Detailed Echocardiographic Phenotyping in Breast Cancer Patients: Associations With Ejection Fraction Decline, Recovery, and Heart Failure Symptoms Over 3 Years of Follow-Up. *Circulation* 2017;135(15):1397-412.

43. Lin H, Guo Y, Zheng Y, et al. Long-Term Effects of Ambient PM2.5 on Hypertension and Blood Pressure and Attributable Risk Among Older Chinese Adults. *Hypertension (Dallas, Tex : 1979)* 2017;69(5):806-12.

44. Xing CY, Tarumi T, Meijers RL, et al. Arterial Pressure, Heart Rate, and Cerebral Hemodynamics Across the Adult Life Span. *Hypertension (Dallas, Tex : 1979)* 2017;69(4):712-20.

45. Benschop L, Schalekamp-Timmermans S, Broere-Brown ZA, et al. Placental Growth Factor as an Indicator of Maternal Cardiovascular Risk After Pregnancy. *Circulation* 2019;139(14):1698-709.

46. Vasan SK, Noordam R, Gowri MS, et al. The proposed systemic thermogenic metabolites succinate and 12,13-diHOME are inversely associated with adiposity and related metabolic traits: evidence from a large human cross-sectional study. *Diabetologia* 2019.

47. Simons N, Dekker JM, van Greevenbroek MM, et al. A Common Gene Variant in Glucokinase Regulatory Protein Interacts With Glucose Metabolism on Diabetic Dyslipidemia: the Combined CODAM and Hoorn Studies. *Diabetes care* 2016;39(10):1811-7.

48. Wolf K, Popp A, Schneider A, et al. Association Between Long-term Exposure to Air Pollution and Biomarkers Related to Insulin Resistance, Subclinical Inflammation, and Adipokines. *Diabetes* 2016;65(11):3314-26.

49. Much D, Beyerlein A, Kindt A, et al. Lactation is associated with altered metabolomic signatures in women with gestational diabetes. *Diabetologia* 2016;59(10):2193-202.

50. Retnakaran R, Ye C, Kramer CK, et al. Maternal Serum Prolactin and Prediction of Postpartum beta-Cell Function and Risk of Prediabetes/Diabetes. *Diabetes care* 2016;39(7):1250-8.

51. Weber KS, Nowotny B, Strassburger K, et al. The Role of Markers of Low-Grade Inflammation for the Early Time Course of Glycemic Control, Glucose Disappearance Rate, and beta-Cell Function in Recently Diagnosed Type 1 and Type 2 Diabetes. *Diabetes care* 2015;38(9):1758-67.

52. Lee CC, Watkins SM, Lorenzo C, et al. Branched-Chain Amino Acids and Insulin Metabolism: The Insulin Resistance Atherosclerosis Study (IRAS). *Diabetes care* 2016;39(4):582-8.

53. Mai XM, Videm V, Sheehan NA, et al. Potential causal associations of serum 25-hydroxyvitamin D with lipids: a Mendelian randomization approach of the HUNT study. *European journal of epidemiology* 2019;34(1):57-66.

54. Orho-Melander M, Hindy G, Borgquist S, et al. Blood lipid genetic scores, the HMGCR gene and cancer risk: a Mendelian randomization study. *International journal of epidemiology* 2018;47(2):495-505.

55. Whitaker KM, Buman MP, Odegaard AO, et al. Sedentary Behaviors and Cardiometabolic Risk: An Isotemporal Substitution Analysis. *American journal of epidemiology* 2018;187(2):181-9.

56. Huang JY, Gavin AR, Richardson TS, et al. Accounting for Life-Course Exposures in Epigenetic Biomarker Association Studies: Early Life Socioeconomic Position, Candidate Gene DNA Methylation, and Adult Cardiometabolic Risk. *American journal of epidemiology* 2016;184(7):520-31.

57. Vogt S, Wahl S, Kettunen J, et al. Characterization of the metabolic profile associated with serum 25-hydroxyvitamin D: a cross-sectional analysis in population-based data. *International journal of epidemiology* 2016;45(5):1469-81.

58. Wang Q, Wurtz P, Auro K, et al. Effects of hormonal contraception on systemic metabolism: cross-sectional and longitudinal evidence. *International journal of epidemiology* 2016;45(5):1445-57.

59. Cabrera SE, Mindell JS, Toledo M, et al. Associations of Blood Pressure With Geographical Latitude, Solar Radiation, and Ambient Temperature: Results From the Chilean Health Survey, 2009-2010. *American journal of epidemiology* 2016;183(11):1071-3.

60. Shanley RP, Hayes RB, Cromar KR, et al. Particulate Air Pollution and Clinical Cardiovascular Disease Risk Factors. *Epidemiology (Cambridge, Mass)* 2016;27(2):291-8.

61. Ferguson TS, Younger-Coleman NO, Tulloch-Reid MK, et al. Birth weight and maternal socioeconomic circumstances were inversely related to systolic blood pressure among Afro-Caribbean young adults. *Journal of clinical epidemiology* 2015;68(9):1002-9.

62. Beeghly-Fadiel A, Khankari NK, Delahanty RJ, et al. A Mendelian randomization analysis of circulating lipid traits and breast cancer risk. *International journal of epidemiology* 2019.

63. van der Schaft N, Schoufour JD, Nano J, et al. Dietary antioxidant capacity and risk of type 2 diabetes mellitus, prediabetes and insulin resistance: the Rotterdam Study. *European journal of epidemiology* 2019;34(9):853-61.

64. Gnatiuc L, Alegre-Diaz J, Halsey J, et al. Adiposity and Blood Pressure in 110 000 Mexican Adults. *Hypertension (Dallas, Tex : 1979)* 2017;69(4):608-14.

65. Han L, Duan D, Zhang S, et al. Effects of the interaction between glycated haemoglobin genetic risk score and postpartum weight reduction on glycaemic changes: A gene-weight interaction analysis. *Diabetes, obesity & metabolism* 2018;20(12):2733-9.

66. Swindell N, Mackintosh K, McNarry M, et al. Objectively Measured Physical Activity and Sedentary Time Are Associated With Cardiometabolic Risk Factors in Adults With Prediabetes: The PREVIEW Study. *Diabetes care* 2018;41(3):562-9.

67. Meeks KAC, Henneman P, Venema A, et al. Epigenome-wide association study in whole blood on type 2 diabetes among sub-Saharan African individuals: findings from the RODAM study. *International journal of epidemiology* 2019;48(1):58-70.

68. Wurtz P, Wang Q, Niironen M, et al. Metabolic signatures of birthweight in 18 288 adolescents and adults. *International journal of epidemiology* 2016;45(5):1539-50.

69. Chen Z, Smith M, Du H, et al. Blood pressure in relation to general and central adiposity among 500 000 adult Chinese men and women. *International journal of epidemiology* 2015;44(4):1305-19.

70. Strand LB, Carnethon M, Biggs ML, et al. Sleep Disturbances and Glucose Metabolism in Older Adults: The Cardiovascular Health Study. *Diabetes care* 2015;38(11):2050-8.

71. Lidegaard LP, Hansen AL, Johansen NB, et al. Physical activity energy expenditure vs cardiorespiratory fitness level in impaired glucose metabolism. *Diabetologia* 2015;58(12):2709-17.

72. Huang T, Zeleznik OA, Poole EM, et al. Habitual sleep quality, plasma metabolites and risk of coronary heart disease in post-menopausal women. *International journal of epidemiology* 2018.

73. Ren R, Covassin N, Yang L, et al. Objective but Not Subjective Short Sleep Duration Is Associated With Hypertension in Obstructive Sleep Apnea. *Hypertension (Dallas, Tex : 1979)* 2018;72(3):610-7.

74. Sun D, Li X, Heianza Y, et al. History of Asthma From Childhood and Arterial Stiffness in Asymptomatic Young Adults: The Bogalusa Heart Study. *Hypertension (Dallas, Tex : 1979)* 2018;71(5):928-36.

75. Lew J, Sanghavi M, Ayers CR, et al. Sex-Based Differences in Cardiometabolic Biomarkers. *Circulation* 2017;135(6):544-55.

76. Yano Y, Fujimoto S, Kramer H, et al. Long-Term Blood Pressure Variability, New-Onset Diabetes Mellitus, and New-Onset Chronic Kidney Disease in the Japanese General Population. *Hypertension (Dallas, Tex : 1979)* 2015;66(1):30-6.

77. van Eupen MG, Schram MT, van Sloten TT, et al. Skin Autofluorescence and Pentosidine Are Associated With Aortic Stiffening: The Maastricht Study. *Hypertension (Dallas, Tex : 1979)* 2016;68(4):956-63.

78. Ruiz-Hurtado G, Ruilope LM, de la Sierra A, et al. Association Between High and Very High Albuminuria and Nighttime Blood Pressure: Influence of Diabetes and Chronic Kidney Disease. *Diabetes care* 2016;39(10):1729-37.

79. Brouwer A, van Raalte DH, Rutters F, et al. Sleep and HbA(1c) in Patients With Type 2 Diabetes: Which Sleep Characteristics Matter Most? *Diabetes care* 2020;43(1):235-43.

80. Curto A, Wellenius GA, Milà C, et al. Ambient Particulate Air Pollution and Blood Pressure in Peri-urban India. *Epidemiology (Cambridge, Mass)* 2019;30(4):492-500.

81. Pazoki R, Dehghan A, Evangelou E, et al. Genetic Predisposition to High Blood Pressure and Lifestyle Factors: Associations With Midlife Blood Pressure Levels and Cardiovascular Events. *Circulation* 2018;137(7):653-61.

82. Parikh NI, Norberg M, Ingelsson E, et al. Association of Pregnancy Complications and Characteristics With Future Risk of Elevated Blood Pressure: The Vasterbotten Intervention Program. *Hypertension (Dallas, Tex : 1979)* 2017;69(3):475-83.

83. Scannell Bryan M, Sofer T, Mossavar-Rahmani Y, et al. Mendelian randomization of inorganic arsenic metabolism as a risk factor for hypertension- and diabetes-related traits among adults in the Hispanic Community Health Study/Study of Latinos (HCHS/SOL) cohort. *Int J Epidemiol* 2019;48(3):876-86.

84. Buglioni A, Cannone V, Cataliotti A, et al. Circulating aldosterone and natriuretic peptides in the general community: relationship to cardiorenal and metabolic disease. *Hypertension (Dallas, Tex : 1979)* 2015;65(1):45-53.

85. Heida KY, Franx A, van Rijn BB, et al. Earlier Age of Onset of Chronic Hypertension and Type 2 Diabetes Mellitus After a Hypertensive Disorder of Pregnancy or Gestational Diabetes Mellitus. *Hypertension (Dallas, Tex : 1979)* 2015;66(6):1116-22.

86. Zhao Q, Zmuda JM, Kuipers AL, et al. Muscle Attenuation Is Associated With Newly Developed Hypertension in Men of African Ancestry. *Hypertension (Dallas, Tex : 1979)* 2017;69(5):957-63.

87. Climie RE, Boutouyrie P, Perier MC, et al. Association Between Occupational, Sport, and Leisure Related Physical Activity and Baroreflex Sensitivity: The Paris Prospective Study III. *Hypertension (Dallas, Tex : 1979)* 2019;74(6):1476-83.

88. Seven E, Thuesen BH, Linneberg A, et al. Abdominal Adiposity Distribution Quantified by Ultrasound Imaging and Incident Hypertension in a General Population. *Hypertension (Dallas, Tex : 1979)* 2016;68(5):1115-22.

89. Chang Y, Kim JH, Noh JW, et al. Prostate-Specific Antigen Within the Reference Range, Subclinical Coronary Atherosclerosis, and Cardiovascular Mortality. *Circulation research* 2019;124(10):1492-504.

90. Moulton CD, Pickup JC, Rokakis AS, et al. The Prospective Association Between Inflammation and Depressive Symptoms in Type 2 Diabetes Stratified by Sex. *Diabetes care* 2019;42(10):1865-72.

91. Foussard N, Cougnard-Gregoire A, Rajaobelina K, et al. Skin Autofluorescence of Pregnant Women With Diabetes Predicts the Macrosomia of Their Children. *Diabetes* 2019;68(8):1663-9.

92. Sasongko MB, Widyaputri F, Sulistyoningrum DC, et al. Estimated Resting Metabolic Rate and Body Composition Measures Are Strongly Associated With Diabetic Retinopathy in Indonesian Adults With Type 2 Diabetes. *Diabetes care* 2018;41(11):2377-84.

93. Lu J, Ma X, Zhou J, et al. Association of Time in Range, as Assessed by Continuous Glucose Monitoring, With Diabetic Retinopathy in Type 2 Diabetes. *Diabetes care* 2018;41(11):2370-6.

94. Castelblanco E, Hernandez M, Castelblanco A, et al. Low-grade Inflammatory Marker Profile May Help to Differentiate Patients With LADA, Classic Adult-Onset Type 1 Diabetes, and Type 2 Diabetes. *Diabetes care* 2018;41(4):862-8.

95. Saulnier PJ, Gand E, Velho G, et al. Association of Circulating Biomarkers (Adrenomedullin, TNFR1, and NT-proBNP) With Renal Function Decline in Patients With Type 2 Diabetes: A French Prospective Cohort. *Diabetes care* 2017;40(3):367-74.

96. Hunt KJ, Baker NL, Cleary PA, et al. Longitudinal Association Between Endothelial Dysfunction, Inflammation, and Clotting Biomarkers With Subclinical Atherosclerosis in Type 1 Diabetes: An Evaluation of the DCCT/EDIC Cohort. *Diabetes care* 2015;38(7):1281-9.

97. Xanthakis V, Sung JH, Samdarshi TE, et al. Relations between subclinical disease markers and type 2 diabetes, metabolic syndrome, and incident cardiovascular disease: the Jackson Heart Study. *Diabetes care* 2015;38(6):1082-8.

98. Ko KP, Kim CS, Ahn Y, et al. Plasma isoflavone concentration is associated with decreased risk of type 2 diabetes in Korean women but not men: results from the Korean Genome and Epidemiology Study. *Diabetologia* 2015;58(4):726-35.

99. Herrmann M, Sullivan DR, Veillard AS, et al. Serum 25-hydroxyvitamin D: a predictor of macrovascular and microvascular complications in patients with type 2 diabetes. *Diabetes care* 2015;38(3):521-8.

100. Sink KM, Divers J, Whitlow CT, et al. Cerebral structural changes in diabetic kidney disease: African American-Diabetes Heart Study MIND. *Diabetes care* 2015;38(2):206-12.

101. Jones S, Tillin T, Williams S, et al. Type 2 diabetes does not account for ethnic differences in exercise capacity or skeletal muscle function in older adults. *Diabetologia* 2020;63(3):624-35.

102. Florido R, Lee AK, McEvoy JW, et al. Cancer Survivorship and Subclinical Myocardial Damage: The Atherosclerosis Risk in Communities (ARIC) Study. *American journal of epidemiology* 2019.

103. Elovainio M, Sommerlad A, Hakulinen C, et al. Structural social relations and cognitive ageing trajectories: evidence from the Whitehall II cohort study. *International journal of epidemiology* 2017.

104. Su TC, Hwang JJ, Yang YR, et al. Association Between Long-term Exposure to Traffic-related Air Pollution and Inflammatory and Thrombotic Markers in Middle-aged Adults. *Epidemiology (Cambridge, Mass)* 2017;28 Suppl 1:S74-s81.

105. Qiu G, Zheng Y, Wang H, et al. Plasma metabolomics identified novel metabolites associated with risk of type 2 diabetes in two prospective cohorts of Chinese adults. *International journal of epidemiology* 2016;45(5):1507-16.

106. Myte R, Gylling B, Schneede J, et al. Components of One-carbon Metabolism Other than Folate and Colorectal Cancer Risk. *Epidemiology (Cambridge, Mass)* 2016;27(6):787-96.

107. Huo X, Gao L, Guo L, et al. Risk of non-fatal cardiovascular diseases in early-onset versus late-onset type 2 diabetes in China: a cross-sectional study. *The lancet Diabetes & endocrinology* 2016;4(2):115-24.

108. Faillie JL, Filion KB, Patenaude V, et al. Dipeptidyl peptidase-4 inhibitors and the risk of community-acquired pneumonia in patients with type 2 diabetes. *Diabetes, obesity & metabolism* 2015;17(4):379-85.

109. Tomiyama H, Shiina K, Vlachopoulos C, et al. Involvement of Arterial Stiffness and Inflammation in Hyperuricemia-Related Development of Hypertension. *Hypertension (Dallas, Tex : 1979)* 2018;72(3):739-45.

110. Laird EJ, McNicholas T, O'Halloran AM, et al. Vitamin D Status Is Not Associated With Orthostatic Hypotension in Older Adults. *Hypertension (Dallas, Tex : 1979)* 2019;74(3):639-44.

111. Wu J, Hall M, Dondo TB, et al. Association between time of hospitalization with acute myocardial infarction and in-hospital mortality. *European heart journal* 2019;40(15):1214-21.

112. Bano A, Chaker L, Mattace-Raso FUS, et al. Thyroid Function and the Risk of Atherosclerotic Cardiovascular Morbidity and Mortality: The Rotterdam Study. *Circulation research* 2017;121(12):1392-400.

113. Guo J, Fujiyoshi A, Willcox B, et al. Increased Aortic Calcification Is Associated With Arterial Stiffness Progression in Multiethnic Middle-Aged Men. *Hypertension (Dallas, Tex : 1979)* 2017;69(1):102-8.

114. Dubin RF, Guajardo I, Ayer A, et al. Associations of Macro- and Microvascular Endothelial Dysfunction With Subclinical Ventricular Dysfunction in End-Stage Renal Disease. *Hypertension (Dallas, Tex : 1979)* 2016;68(4):913-20.

115. van der Veen PH, Geerlings MI, Visseren FL, et al. Hypertensive Target Organ Damage and Longitudinal Changes in Brain Structure and Function: The Second Manifestations of Arterial Disease-Magnetic Resonance Study. *Hypertension (Dallas, Tex : 1979)* 2015;66(6):1152-8.

116. Vergoossen LW, Schram MT, de Jong JJ, et al. White Matter Connectivity Abnormalities in Prediabetes and Type 2 Diabetes: The Maastricht Study. *Diabetes care* 2019.

117. Ozcan B, Rutters F, Snoek FJ, et al. High Diabetes Distress Among Ethnic Minorities Is Not Explained by Metabolic, Cardiovascular, or Lifestyle Factors: Findings From the Dutch Diabetes Pearl Cohort. *Diabetes care* 2018;41(9):1854-61.

118. Gordin D, Harjutsalo V, Tinsley L, et al. Differential Association of Microvascular Attributions With Cardiovascular Disease in Patients With Long Duration of Type 1 Diabetes. *Diabetes care* 2018;41(4):815-22.

119. Bermingham ML, Colombo M, McGurnaghan SJ, et al. N-Glycan Profile and Kidney Disease in Type 1 Diabetes. *Diabetes care* 2018;41(1):79-87.

120. Ljungman PL, Wilker EH, Rice MB, et al. The Impact of Multipollutant Clusters on the Association Between Fine Particulate Air Pollution and Microvascular Function. *Epidemiology (Cambridge, Mass)* 2016;27(2):194-201.

121. Endes S, Schaffner E, Caviezel S, et al. Physical activity is associated with lower arterial stiffness in older adults: results of the SAPALDIA 3 Cohort Study. *European journal of epidemiology* 2016;31(3):275-85.

122. Boczar KE, Cheung K, Boodhwani M, et al. Sex Differences in Thoracic Aortic Aneurysm Growth. *Hypertension (Dallas, Tex : 1979)* 2019;73(1):190-6.

123. Nanba K, Vaidya A, Williams GH, et al. Age-Related Autonomous Aldosteronism. *Circulation* 2017;136(4):347-55.

124. Ambrosy AP, Parzynski CS, Friedman DJ, et al. Is Time From Last Hospitalization for Heart Failure to Placement of a Primary Prevention Implantable Cardioverter-Defibrillator Associated With Patient Outcomes? *Circulation* 2018;138(24):2787-97.

125. Jefferson AL, Cambronero FE, Liu D, et al. Higher Aortic Stiffness Is Related to Lower Cerebral Blood Flow and Preserved Cerebrovascular Reactivity in Older Adults. *Circulation* 2018;138(18):1951-62.

126. Ohno Y, Sone M, Inagaki N, et al. Prevalence of Cardiovascular Disease and Its Risk Factors in Primary Aldosteronism: A Multicenter Study in Japan. *Hypertension (Dallas, Tex : 1979)* 2018;71(3):530-7.

127. James SN, Wong A, Tillin T, et al. The effect of mid-life insulin resistance and type 2 diabetes on older-age cognitive state: the explanatory role of early-life advantage. *Diabetologia* 2019;62(10):1891-900.
