# Supplementary material for: How measurements affected by medication use are reported and handled in observational research: A literature review
Source: Pharmacoepidemiol Drug Saf. 2022 May 4;31(7):739–48. doi: 10.1002/pds.5437 (PMC9321697; doi:10.1002/pds.5437)
Supplement: Supplementary file 4 — Appendix S4: Supporting information. [file PDS-31-739-s003.docx]

**Supplementary material 4. Methods used for sensitivity analyses**

| **Affected variable** | **First author** | **Main analysis method** | **Sensitivity analysis method** | **Journal field** |
| --- | --- | --- | --- | --- |
| **Exposure** | Fan et al. (1) | Adjusting for using medication | Restricting to individuals free of a condition | Cardiology |
|  | Mahinrad et al. (2) | Ignoring | Restricting to non-medication users | Cardiology |
|  | Glodzik et al. (3) | Ignoring | Restricting to individuals free of a condition | Cardiology |
|  |  |  | Restricting to individuals having a condition |  |
|  | James et al. (4) | Ignoring | Adjusting for having a condition (yes/no) | Diabetes |
|  |  |  | Replace to maximum values within the cohort |  |
|  | Punthakee et al. (5) | Adjusting for using medication | Restricting to individuals free of a condition | Diabetes |
|  |  |  | Restricting to individuals with a condition |  |
|  | Parrinello et al. (6) | Ignoring | Restricting to individuals free of diabetes | Diabetes |
| **Outcome** | Benschop et al. (7) | Restricting to individuals free of a condition at the baseline | Restricting to individuals free of a condition during the entire follow-up | Cardiology |
|  | Pazoki et al. (8) | Adding medication effect | Restricting to non-medication users | Cardiology |
|  | Cai et al. (9) | Ignoring | Adjusting for using medication (yes/no) | Cardiology |
|  | Yano et al. (10) | Adjusting for using medication | Restricting to individuals free of a condition | Cardiology |
|  | Koivistoinen et al. (11) | Ignoring | Restricting to individuals free of a condition | Cardiology |
|  |  |  | Restricting to individuals free of a condition |  |
|  | Wolf et al. (12) | Ignoring | Restricting to non-medication users | Diabetes |
|  | Much et al. (13) | Ignoring | Adjusting for using medication (yes/no) | Diabetes |
|  | Bonnefond et al. (14) | Ignoring | Adjusting for using medication (yes/no) | Diabetes |
|  | Weber et al. (15) | Ignoring | Restricting to non-medication users | Diabetes |
|  | Simons et al. (16) | Ignoring | Restricting to non-medication users | Diabetes |
|  | Cabrera et al. (17) | Ignoring | Restricting to non-medication users | Epidemiology |

**Appendix 4. (continued)**

| **Affected variable** | **First author** | **Main analysis method** | **Sensitivity analysis method** | **Journal field** |
| --- | --- | --- | --- | --- |
| **Outcome** | Chen et al. (18) | Restricting to non-medication users | Restricting to medication users | Epidemiology |
|  | Scannell et al. (19) | Adding a constant value | Adjusting for using medication (yes/no) | Epidemiology |
|  | Vogt et al. (20) | Ignoring | Adjusting for using medication (yes/no) | Epidemiology |
|  | Curto et al. (21) | Adjusting for using medication | Restricting to non-medication users | Epidemiology |
| **Confounder** | Buglioni et al. (22) | Ignoring | Restricting to non-medication users | Cardiology |
|  |  |  | Restricting to medication users |  |

1. Fan F, Qi L, Jia J, et al. Noninvasive Central Systolic Blood Pressure Is More Strongly Related to Kidney Function Decline Than Peripheral Systolic Blood Pressure in a Chinese Community-Based Population. *Hypertension (Dallas, Tex : 1979)* 2016;67(6):1166-72.

2. Mahinrad S, Kurian S, Garner CR, et al. Cumulative Blood Pressure Exposure During Young Adulthood and Mobility and Cognitive Function in Midlife. *Circulation* 2020;141(9):712-24.

3. Glodzik L, Rusinek H, Tsui W, et al. Different Relationship Between Systolic Blood Pressure and Cerebral Perfusion in Subjects With and Without Hypertension. *Hypertension (Dallas, Tex : 1979)* 2019;73(1):197-205.

4. James SN, Wong A, Tillin T, et al. The effect of mid-life insulin resistance and type 2 diabetes on older-age cognitive state: the explanatory role of early-life advantage. *Diabetologia* 2019;62(10):1891-900.

5. Punthakee Z, Iglesias PP, Alonso-Coello P, et al. Association of preoperative glucose concentration with myocardial injury and death after non-cardiac surgery (GlucoVISION): a prospective cohort study. *The lancet Diabetes & endocrinology* 2018;6(10):790-7.

6. Parrinello CM, Sharrett AR, Maruthur NM, et al. Racial Differences in and Prognostic Value of Biomarkers of Hyperglycemia. *Diabetes Care* 2016;39(4):589-95.

7. Benschop L, Schalekamp-Timmermans S, Broere-Brown ZA, et al. Placental Growth Factor as an Indicator of Maternal Cardiovascular Risk After Pregnancy. *Circulation* 2019;139(14):1698-709.

8. Pazoki R, Dehghan A, Evangelou E, et al. Genetic Predisposition to High Blood Pressure and Lifestyle Factors: Associations With Midlife Blood Pressure Levels and Cardiovascular Events. *Circulation* 2018;137(7):653-61.

9. Cai Y, Hansell AL, Blangiardo M, et al. Long-term exposure to road traffic noise, ambient air pollution, and cardiovascular risk factors in the HUNT and lifelines cohorts. *European heart journal* 2017;38(29):2290-6.

10. Yano Y, Fujimoto S, Kramer H, et al. Long-Term Blood Pressure Variability, New-Onset Diabetes Mellitus, and New-Onset Chronic Kidney Disease in the Japanese General Population. *Hypertension (Dallas, Tex : 1979)* 2015;66(1):30-6.

11. Koivistoinen T, Lyytikainen LP, Aatola H, et al. Pulse Wave Velocity Predicts the Progression of Blood Pressure and Development of Hypertension in Young Adults. *Hypertension (Dallas, Tex : 1979)* 2018;71(3):451-6.

12. Wolf K, Popp A, Schneider A, et al. Association Between Long-term Exposure to Air Pollution and Biomarkers Related to Insulin Resistance, Subclinical Inflammation, and Adipokines. *Diabetes* 2016;65(11):3314-26.

13. Much D, Beyerlein A, Kindt A, et al. Lactation is associated with altered metabolomic signatures in women with gestational diabetes. *Diabetologia* 2016;59(10):2193-202.

14. Bonnefond A, Yengo L, Le May C, et al. The loss-of-function PCSK9 p.R46L genetic variant does not alter glucose homeostasis. *Diabetologia* 2015;58(9):2051-5.

15. Weber KS, Nowotny B, Strassburger K, et al. The Role of Markers of Low-Grade Inflammation for the Early Time Course of Glycemic Control, Glucose Disappearance Rate, and beta-Cell Function in Recently Diagnosed Type 1 and Type 2 Diabetes. *Diabetes care* 2015;38(9):1758-67.

16. Simons N, Dekker JM, van Greevenbroek MM, et al. A Common Gene Variant in Glucokinase Regulatory Protein Interacts With Glucose Metabolism on Diabetic Dyslipidemia: the Combined CODAM and Hoorn Studies. *Diabetes care* 2016;39(10):1811-7.

17. Cabrera SE, Mindell JS, Toledo M, et al. Associations of Blood Pressure With Geographical Latitude, Solar Radiation, and Ambient Temperature: Results From the Chilean Health Survey, 2009-2010. *American journal of epidemiology* 2016;183(11):1071-3.

18. Chen Z, Smith M, Du H, et al. Blood pressure in relation to general and central adiposity among 500 000 adult Chinese men and women. *International journal of epidemiology* 2015;44(4):1305-19.

19. Scannell Bryan M, Sofer T, Mossavar-Rahmani Y, et al. Mendelian randomization of inorganic arsenic metabolism as a risk factor for hypertension- and diabetes-related traits among adults in the Hispanic Community Health Study/Study of Latinos (HCHS/SOL) cohort. *Int J Epidemiol* 2019;48(3):876-86.

20. Vogt S, Wahl S, Kettunen J, et al. Characterization of the metabolic profile associated with serum 25-hydroxyvitamin D: a cross-sectional analysis in population-based data. *International journal of epidemiology* 2016;45(5):1469-81.

21. Curto A, Wellenius GA, Milà C, et al. Ambient Particulate Air Pollution and Blood Pressure in Peri-urban India. *Epidemiology (Cambridge, Mass)* 2019;30(4):492-500.

22. Buglioni A, Cannone V, Cataliotti A, et al. Circulating aldosterone and natriuretic peptides in the general community: relationship to cardiorenal and metabolic disease. *Hypertension (Dallas, Tex : 1979)* 2015;65(1):45-53.
